# Cognition of diet quality and dietary management in elderly patients with coronary and other atherosclerotic vascular disease in western China, a qualitative research study

**DOI:** 10.1186/s12877-024-05058-2

**Published:** 2024-06-17

**Authors:** Jiamengying Chen, Xiaojie Li, Yun Wang, Chunling Zhang, Li Yang, Lvheng Zhao, Qingqing Zhu, Li Wang, Yixia Zhou

**Affiliations:** 1 Nursing School, Guizhou University of Traditional Chinese Medicine, Guiyang City, Guizhou Province China; 2 Nursing School, Guizhou Medical University, Guiyang City, Guizhou Province China; 3 The Second Affiliated Hospital, Guizhou University of Traditional Chinese Medicine, Guiyang City, Guizhou Province China; 4 School of Nursing, Suzhou Medical College of Soochow University, Suzhou City, Jiangsu Province China

**Keywords:** Atherosclerosis, Nutritional assistance, Diet, Qualitative research

## Abstract

**Background:**

Healthy eating is one of the most important nonpharmacologic treatments for patients with atherosclerosis(AS). However, it is unclear how elderly AS patients in western China perceive their dietary status and which type of nutritional assistance they would be willing to receive. Therefore, the primary purpose of this study was to understand the level of knowledge about current dietary habits and healthy eating habits among elderly AS patients in western China, and the secondary purpose was to identify acceptable nutritional assistance measures or pathways for those patients to help them manage disease progression.

**Methods:**

An implementation study approach was used to recruit elderly patients with AS-related diseases in western China for semistructured interviews.

**Results:**

14 participants were included in the study, and the following three themes were identified from the interviews:(1) the diet with regional characteristics; (2) low nutrition-related health literacy; (3) complex attitudes towards nutritional assistance. Most participants had misconceptions about healthy eating, and the sources of their knowledge might not be trustworthy. Participants expressed a preference for personalized nutritional assistance, especially that provided by medical-nursing combined institutions.

**Conclusion:**

Patients in western China need nutritional assistance for their regional dietary habits; therefore, healthy dietary patterns consistent with the regional culture are proposed to improve the prevailing lack of knowledge about healthy diets, improve the dietary structure of patients, and control the development of the disease.

**Supplementary Information:**

The online version contains supplementary material available at 10.1186/s12877-024-05058-2.

## Background

Patients generally misunderstand dietary information, and their perceptions of dietary quality are different. With improvements in people’s living standards and a general lack of exercise, the incidence of atherosclerosis (AS) is increasing annually. The main incidence group is still the elderly population [1], and this disease has brought a greater economic burden to people and medical systems [2].

Poor eating habits are a definite risk factor for AS and one of the important risk factors associated with the burden of cardiovascular disease (CVD) [3]. In 2016, 2.1 million global deaths from CVD were linked to poor eating habits [4]. Many studies had shown that most people with AS have poor diet quality and poor knowledge of healthy diets [5]. Global comparative risk assessment studies have estimated that hundreds of thousands or even millions of deaths in patients with CVD can be attributed to the effects of certain diets and environments [6]. In China, many scholars had investigated the dietary behaviour of patients with AS. With the further development of the economy and the steady increase in the degree of urbanization [7], Chinese consumption of fruits, dairy products, snacks, fast food and beverages is increasing significantly, and the dietary pattern is gradually shifting to a high-fat Western diet [8]. This tendency may be closely related to the increasing incidence of AS-related diseases. China is a vast country, which leads to different eating habits among people in different regions. A study of 11,512 respondents in 47 provinces of China showed that the mortality rate of CVD in the central and western regions was greater than that in the eastern provinces of China, and poor eating habits were one of the risk factors for death. However, we found that the current research is still targeting individuals living in the eastern and northern regions of China [9]. There is a lack of surveys on people in western China, which may lead to a lack of targeted and personalized nutritional assistance for this population [10].

Nutritional assistance methods include providing relevant dietary advice [11], diet intervention measures [12], diet patterns [13], nutritional supplements [14], etc. In previous studies, health education related to diet management has been shown to effectively improve the disease awareness of patients with AS and to have a positive impact on some of its indicators, such as blood lipid levels and body mass index [15]. Before designing interventions, some investigators did not consider whether participants were willing to accept nutritional assistance, and they lacked an understanding of the participants’ daily life [16]. Moreover, researchers and clinical staff may be biased against interventions recognized by patients [17]. The incorporation of the perspective of patients can help researchers explore new interventions or discover new understandings of existing interventions to form higher-quality research. Understanding local eating habits in advance can also help researchers better identify the possible bad eating behaviours of the target group and develop more targeted interventions [18].

The main purpose of this study was to explore the views of patients with coronary and other atherosclerotic vascular diseases in western China on dietary quality and previously received dietary recommendations or nutritional assistance. The secondary purpose was to determine which nutritional assistance methods are acceptable for these patients to help them improve their health management.

## Methods

### Qualitative approach & research paradigm

This was a qualitative study, and we used a semistructured interview method. Mainly, we discussed how patients with coronary and other atherosclerotic vascular diseases viewed their dietary habits and intake, as well as their views on various nutritional assistance methods and approaches, and explored their feelings and expectations regarding nutritional assistance.

### Researcher characteristics and reflexivity

Two researchers (Li Wang, Yixia Zhou) were responsible for the research design, and 1 researcher (Li Yang) who had a clinical nurse–patient relationship with the interviewees recruited and screened participants with the assistance of 3 researchers (Lvheng Zhao, Qingqing Zhu, Yun Wang). Two researchers (Jiamengying Chen, Xiaojie Li) conducted patient interviews under the supervision of a nutrition expert (Chunling Zhang) and entered and analysed the data. A total of 9 researchers participated in this study, all of whom had research/work backgrounds related to nutrition or CVD.

### Context

From March 2023 to June 2023, elderly people who visited 3 medical institutions in Guizhou Province, China, were selected as interviewees using purposive sampling methods. The average number of elderly people in the 3 medical institutions is approximately 80 per week. A stable medical team provides medical security and regularly carries out cardiac rehabilitation and other services.

### Sampling strategy

The inclusion criteria for patients were as follows: (1) $\ge$60 years old; (2) diagnosed with coronary or other atherosclerotic vascular disease [19]; (3) clear thinking, able to speak Chinese fluently, including Mandarin or dialect; and (4) signed written informed consent form to voluntarily participate in the study. The exclusion criteria were as follows: (1) cognitive impairment, (2) communication barriers.

After ethical review, posters were placed in cardiovascular clinics and nutrition clinics of medical institutions to recruit volunteers to participate in the study. Information on the poster included the purpose of the study, inclusion and exclusion criteria, and contact information for the principal investigator (Jiamengying Chen, Xiaojie Li). The posters were posted from February 2023 to May 2023, and 16 elderly patients with AS were invited to participate. Due to data saturation, a total of 14 elderly patients with AS were finally interviewed and numbered P1 to P14.

Before beginning the study, the researchers invited potential participants, explained the purpose and methods of the study to the participants who were willing to participate in the study, and interviewed the participants with their consent.

### Ethical issues pertaining to human subjects

Before the start of the study, the research team provided written informed consent forms to the eligible participants. This study was approved by the the Ethics Committee of The Second Affiliated Hospital of Guizhou University of Traditional Chinese Medicine (No.: KYW2022007).

### Data collection and instruments

Participants participated in research interviews from March 2023 to June 2023. The interviews were conducted in a separate lounge of the medical institutions to ensure participants’ privacy. After obtaining the participants’ consent, the researchers recorded the entire interview, and all recordings were obtained using the same electronic device. All participants were interviewed by the same researcher and supervised by the chief nurses on the research team. The participants had the right to know the educational level, professional title and other information about the researchers.

According to the purpose of the study, the members of the research group conducted a literature review in advance, discussed and formulated the interview outline, and conducted a pre-interview with 2 participants in advance. According to the interview results, the outline was modified, and the interview outline applied in this survey was finally determined. The interview outline consisted of open and closed questions. The main topics of discussion were the participants’ views on the current quality of their diet, whether they feel that their diet should be improved, and whether they were willing to accept medical assistance related to diet management. In addition, the researchers asked participants whether they had received diet-related or nutritionist guidance.

At the end of the interview, the researchers listed many types of nutritional assistance or approaches to participants and asked them to provide preferences for each type of nutritional assistance or approach. Before the interview, the researchers used a warm-up question to create a friendly atmosphere between the interviewer and the interviewee: “If you do not mind, could you tell me something about your AS-related disease?”

### Clinical measures

The researchers collected information such as the participants’ age, sex, and types of disease. This information was collected to provide a sufficient sample description and determine whether there was heterogeneity.

### Units of study

In this study, the saturation of data collection was used as the end point of the interview process; that is, if the data analysis was repeated with the previous data, and no new coding appeared, then the interview process was considered to be completed. After data saturation, 2 participants were interviewed to ensure that no new coding appeared [20]. The interview time ranged from 11 minutes and 08 seconds to 27 minutes and 35 seconds, with an average time of 17 minutes and 42 seconds.

### Data processing

During the interview, the researcher recorded the patient’s intonation, speech rate, expression, gesture and so on. To reduce the researchers’ memory bias, the recordings were converted into text within 24 hours after the end of the interview and supplemented and modified in combination with the notes of on-site observation [21] .

### Data analysis

This study was conducted by 2 researchers (Jiamengying Chen, Xiaojie Li) using the Colaizzi seven-step method of phenomenological research to guide the data analysis. The 2 researchers independently and repeatedly listened to the audio recordings of the interviews, verified the content, and ultimately analysed the data separately.

During the study, the researcher verified unclear statements in the recordings by contacting the respondent via WeChat or telephone. In addition, the transcribed notes and the themes generated from the analysis were confirmed with the interviewees to ensure that their views were authentically recorded. After the information was completed for thematic extraction and coding, the research team held 1 team meeting to review it. All the researchers commented on and ultimately agreed on the themes and coding of the interviews.

## Results

### Participant characteristics

Fourteen elderly patients with atherosclerotic vascular disease, with an average age of 75 years, were included in the study. Five participants were male, and 9 participants were female. The disease categories included coronary atherosclerotic heart disease, cerebral infarction, and carotid atherosclerotic plaque. Participant information is shown in Table 1.

**Table 1:** Basic information of 14 elderly patients with coronary and other atherosclerotic vascular diseases who participated in semi-structured interviews

		Range
Participants(n)	14	/
Mean age(n)	75	61-90
Gender,n(%)		
Gender(n)		
female	9(64.29%)	/
male	5(35.71%)	/
Disease type,n(%)		
coronary atherosclerotic heart disease	7(50.00%)	/
cerebral infarction(cerebral AS)	4(28.57%)	/
carotid atherosclerotic plaque	3(21.43%)	/

### Themes

The results of this study show the acceptability of the current dietary status, the understanding of previous nutritional assistance, and the methods of future nutritional assistance in elderly patients with AS-related diseases in western China. The following 3 themes emerged from this study: (1) the diet with regional characteristics; (2) low nutrition-related health literacy; (3) complex attitudes towards nutritional assistance.

#### The diet with regional characteristics

In terms of staple food preferences, most of the elderly people included in this study claimed that they consumed rice vermicelli for breakfast and lunch because it is “easily digestible” (P3, female, 71 years old). They liked to add animal fats when eating rice vermicelli or noodles (especially ChangWang noodles from Guizhou, China), even if they knew that animal fats can be harmful to the body. These animal fats included solid animal fats and fried animal fats (known as CuiShao) to increase the flavour of the food. Another common breakfast choice among these participants was steamed glutinous rice with chili oil, soy sauce and a variety of side dishes, including “CuiShao”, bacon or sausage, fried peanuts and so on. The family members met the participants’ requests and provided them with this type of food.

*“I eat either rice vermicelli or ChangWang noodles every morning. Sometimes (I) do not want to go downstairs, and I let my son or daughter bring it back to me. I think ChangWang noodles need a lot of “CuiShao” to be delicious.”*

(P14; Male, 73 years old)

Some participants also said that they were not keen on eating refined rice products or noodles but preferred coarse grains, mainly including “corns, sweet potatoes, and potatoes, because this state produces potatoes” (P8; Female, 66 year old). The discussed cooking methods for the potatoes mainly including frying, fire baking and stir-frying.

*“I liked to eat potatoes when I was young, and I also like to eat them now. When I was younger, I would bake my potatoes, but now I prefer fried potatoes.”*

(P12; Male, 81 year old)

Some male participants favoured alcohol. They mainly consumed Chinese Baijiu, but all of them reduced their alcohol consumption after learning that they suffered from AS-related diseases. Female participants widely mentioned that they would like to drink Chinese rice wine (Mijiu) (especially homemade) rather than Chinese Baijiu and considered Chinese rice wine (Mijiu) consumption a habit that “everyone in Guizhou should have” (P9; Female, 83 year old).

*“I used to drink at least 100 ml of Chinese baijiu; after learning that I was sick, I quit drinking.”*

(P13; Male, 64 year old)

Most participants believed that their dietary intake was healthy, while some participants said that after the diagnosis of AS-related diseases, they consciously chose to eat more vegetarian foods, such as ‘Suguadou’, a specialty of Guizhou Province, China, and avoid consuming animal fats.

*“After I got sick, I gained some knowledge from the newspaper and TV. It was said that eating a vegetarian diet is good for my health. [Now] I eat a vegetarian diet and do not eat chicken, duck or fish.”*

(P2; Female, 61 year old)

Other participants said that they liked and frequently ate “red sour soup”, a Chinese Guizhou specialty, 2 to 3 times a week, or even more frequently. They cooked “red sour soup” in dishes by adding water or soup stock and boiled freshwater fish, lean meat and vegetables. They expressed their preference for ethnic-specific eating habits, and even if they chose to eat out, they would more frequently choose restaurants that sell “red sour soup” because “fish is easy to digest for elderly individuals, so we eat fish in sour soup at restaurants, and we like that too” (P13; Male, 64 year old). Some participants expressed their recognition of the simple cooking method of “red sour soup”. Many participants mentioned their decreasing food intake after entering old age, and they indicated that “I cannot eat much, and they say that the amount of one meal I eat is equal to the amount of one meal that a cat eats” (P4; Female, 77 year old), emphasizing “You need to eat something sour to get an appetite” (P3; Female, 71 year old).

*“People in Guizhou should eat red sour soup; I have to eat it several times a week.”*

(P11; Male, 82 year old)

For the intake of fruit, many participants thought that fruit consumption was a treat because their family or caregivers did not allow them to eat too much other food outside of dinner, and being provided with fruit could make them feel happy. “They did not allow me to eat too much fruit, and every time I ate fruit, they were worried that my blood sugar would rise” (P1; Female, 90 year old). The participants usually actively discussed their preferences for fruits, including buying their favourite fruits at the market or asking their caregivers to provide some fruits. Some participants mentioned that they liked to drink *rosa roxburghii Tratt* (RRT) juice or directly ate sliced fresh RRT for “vitamin C supplementation” (P9; Female, 83 year old).

*“This plant [RRT] was widely cultivated in my hometown, and when it was ripe, we picked the fruit and ate it. It became a habit!”*

(P7; Male, 80 year old)

#### Low nutrition-related health literacy

Most of the participants did not receive professional nutritionist consulting services and did not know that the hospital had nutrition-related departments. Some participants mentioned that when visiting a hospital, doctors or nurses mentioned diet-related knowledge, such as avoiding a greasy diet and not eating animal fats, but rarely explained the reasons.

*“Nutrition department? The hospital has this department?” I do not know what to eat, so the doctor told me, ‘eat less oil and less salt.’ However, he did not tell me why”.*

(P3; Female, 71 year old)

The majority of participants stated that they could use the internet to gain much knowledge about healthy eating patterns. In addition to professional notification, participants also obtained diet-related knowledge through newspapers, television, online short video publicity, family notification, etc. “(I) watched many of these kinds of videos on my telephone” (P5; Female, 62 year old). However, they had no way to tell whether the information was correct These information sources contained contradictory content, which made participants unable to distinguish the correctness of the information. Other participants said that they could not learn diet-related knowledge through commonly used health education methods, such as public accounts, videos, and brochures, in tertiary hospitals due to the degradation of vision and hearing caused by age.

*“I’m old, my eyesight is poor, and I cannot see with my glasses! I also want to read the brochure [on nutrition], but I cannot see it clearly”.*

(P12; Male, 81 year old)

Most participants could list the relevant nutritional knowledge they knew, and they also performed a small number of healthy eating behaviours, such as the most basic behaviours: quitting smoking and drinking. They believed that the implementation of a healthy diet contributes to recovery from the diseases.

*“I stopped smoking or drinking after I got sick! I know that these [cigarettes, alcohol] are not good for the body” .*

(P11; Male, 82 year old)

Some participants blindly implemented diet-related knowledge after acquiring it. These participants believed that consuming dietary supplements can ensure good health, so visiting medical institutions was unnecessary. They thought that the greater the intake of dietary supplements, the better the body they would have, even if their health might be harmed by excessive intake.

*“I hardly go to the hospital because I eat a lot of health supplements; my body is fine, and I am fine”.*

(P8; Female, 66 year old).

Although in medical institutions, participants received health education on diet-related knowledge, not all patients were able to effectively implement the information. Some patients were not willing to implement the recommended healthy eating patterns, and they did not want to change their preferences. The participants had different understandings of healthy eating patterns. Some participants were aware of systematic dietary patterns that they described as “good” but “difficult to implement” (P2; Female, 61 year old). Others described these eating patterns as “unpalatable”. A common view is that the ingredients of these dietary patterns are difficult or inaccessible to them.

*“No, no, [they want me to] eat so many vegetables, like I am a rabbit! I have maintained my eating habits for so many years and cannot change them. These diets are weird; I do not eat avocados, I do not eat oats. If I can live to be a hundred years old if I eat these things, then I would rather die at age eighty”.*

(P1; Female, 90 year old)

In addition, many participants said that doctors and nurses could not monitor whether they consumed a healthy diet after leaving hospitals. It is difficult to follow a healthy diet after discharge, especially when most patients and their families do not have a medical background.

*“After I was discharged from the hospital, they [the doctors and nurses] did not know what I was eating at home. Doctors and nurses are very busy with work; how can there be time to help us with our eating?”*

(P13; Male, 64 year old)

#### Complex attitudes towards nutritional assistance

Participants generally expressed fear of diseases. They said, “This disease will stay with me for the rest of my life, and I cannot cure it” (P12; Male, 81 year old). These participants elaborated on their desire to become healthier through nutritional assistance, and they also tended to be more willing to receive dietary-related guidance and assistance and viewed the role of nutritional assistance in delaying the development of AS positively. Personalized nutritional assistance received a positive response from the participants, and they were willing to try nutritional assistance that would help them.

*“I dare not to do anything when I suffer from this disease because I fear that something will happen to my blood vessels..... Of course, it is good to be able to eat healthier; people live to eat three meals a day. If the meal tastes good and the body can be healthy, then I will wake up laughing in my dreams” .*

(P12; Male, 81 year old)

The vast majority of participants expressed their willingness to use customized recipes, diet lists, etc., but the implementation process required the understanding and support of their families. Two male participants said that “My wife is the head of the family”, and whether to use custom recipes and diet lists required the cooperation and consent of his wife. Other patients said that because they are old, whether they could cook according to the recipe required the cooperation of their sons and daughters or caregivers (paid by the elderly individuals themselves or their families).

*“We are all old and need help with daily activities such as eating and dressing. Some things require children’s help to achieve”.*

(P6; Female, 81 year old)

Some participants were not very skilled in the operation of electronic devices such as telephone, computers, or televisions. They also suffered from diseases that caused them to be unable to use communication devices such as telephone. Therefore, they could not receive online health education. They only accepted one-to-one or one-to-many nutritional assistance methods that were held offline. However, some participants mentioned that they would selectively adopt the nutritional recommendations made in the meetings for the public because “not all of them suit me” (P1, female, 90 years old). Other participants suggested that they prefer to use remote online methods for meetings because they “do not have the time or energy to attend the meeting, and it is not safe if the meeting place is far away” (P7; Male, 80 year old); they were worried about traffic safety between hospitals and therefore could not attend the meetings.

*“I am old, and I have no idea how to use telephone or computers for online meetings. So, I prefer offline meetings where we do whatever the doctors and nurses say” .*

(P14; Male, 73 year old).

Some participants were more likely to take dietary supplements such as vitamins rather than considering other forms of nutritional assistance first. Other participants had their own views on dietary supplements; they might try to consume fresh or “medicinal” (P1; Female, 90 year old) ingredients instead of the dietary supplements prescribed by their doctors. Due to the severity of AS-related diseases, these participants were willing to receive various forms of nutritional assistance. Other participants expressed that they had too much concern and distrust regarding the use of dietary supplements. Some participants were worried about the interaction between dietary supplements and the drug treatment they were currently receiving, while other participants thought that were already using too many oral drugs, and whether dietary supplements were useful was uncertain.

*“There are a lot of bad people [selling dietary supplements] now, and it is hard to identify who is good and who is bad”.*

(P12; Male, 81 year old)

Some participants showed the opposite attitude towards nutritional assistance; they believed that they were old enough to receive intervention for their diet. Regarding the malignant cardiovascular events, cerebrovascular events, and amputations that could result from AS-related diseases, these participants stated that they “did not know and did not understand how it could be so serious” (P9; Female, 83 year old).

*“I’m so old, I should eat what I want to eat” .*

(P3; Female, 71 year old)

Most of the participants expressed their willingness to try nutritional assistance measures, which were considered beneficial for delaying the development of AS, including medical-nursing combined institutions that could provide them with a diet, but those facilities put forwards higher requirements on the price and quality of the meals. If they did not meet the requirements, they would not choose this nutritional assistance measure.

*“The community should do something practical for us old people. We will eat what is good, and we do not eat what is bad”.*

(P6; Female, 81 year old)

Some participants said they were concerned about the price of the diet provided by the medical-nursing combined institutions and were worried about their economic situation. When their income was not enough to pay for the diet provided by the medical and nursing institutions, they would not choose this method. Less income had taken away their freedom of consumption.

*“We are all rural people, we have no income, and the cost of eating out is equal to the cost of a few days of our daily life..... If the food is very expensive, we will definitely be unwilling to eat it” .*

(P7; Male, 80 year old)

## Discussion

The results of this study showed the acceptability of the current dietary status, the understanding of previous nutritional assistance, and the methods of future nutritional assistance in elderly patients with AS-related diseases in western China. The theme generated in this study shows that the factors affecting dietary status are multifaceted and complex, and the participants’ dietary preferences had obvious regional characteristics.

The first theme generated by this qualitative research is that the diet with regional characteristics. In this topic, we explored the relationship between participants and their food choices. We found that the participants’ diets had strong regional characteristics, reflecting the regional characteristics of the provinces in western China. The diagnosis of AS-related diseases resulted in some patients changing their eating habits, following the health education of doctors or nurses and choosing to limit alcohol consumption and eat more vegetables. For other participants, there were some difficulties in adhering to healthy eating habits; for example, the tastes and dietary preferences formed during perennial life are difficult to change. The second theme was centred on the implementation of nutritional assistance by participants. We measured participants’ understanding and implantation of knowledge about a healthy diet, which reflected their general misunderstanding of healthy diet knowledge. The third theme was that attitudes towards nutritional assistance were complex; we summarized the participants’ attitudes towards a variety of nutritional assistance approaches. Research has shown that most participants were welcoming and receptive to nutritional assistance, but other patients expressed a resistant attitude. Some participants highlighted their concerns about the price of food.

The participants discussed their current dietary intake with the researchers. In this component of the study, the participants’ dietary preferences showed obvious regionality. This study showed that the mainstream staple food choices for elderly patients with AS-related diseases in western China include rice (including refined rice and its products), glutinous rice, and some coarse grains, such as potatoes and corn. Such staple food choices were suitable for local geographical conditions but might adversely affect the health of participants. Rice products, such as rice vermicelli, were one of the main food choices that participants were interested in. They often mentioned mutton rice vermicelli, beef rice vermicelli, chili chicken rice vermicelli and so on. Most commonly, rice vermicelli and noodles were cooked in boiling water and then put into seasoned broth. Studies have shown that cooked rice flour is a moderate-GI food [22], and a higher GI index has been shown to be significantly associated with an increased risk of CVD [23]. Postprandial hyperglycaemia can lead to elevated triglycerides and increased oxidative stress, which have a negative impact on the vascular endothelium [24].

The participants often mentioned “Cuishao”, bacon, sausage, and fried peanuts. Cuishao is a unique snack and was popular among people living in Guizhou Province, China. Pork (i.e., pork belly meat with more adipose tissue mixed with lean meat) was used as the raw material, and seasonings were added to marinate and then fry the meat. The fried “Cuishao” contained a large amount of oil. Excessive intake of oil can cause a variety of adverse effects on health and may lead to a greater risk of disease, including hypertension, AS and cancer [25]. During the frying process, a series of chemical reactions, such as the oil oxidation reaction, Maillard reaction and oxidative degradation of proteins, occur in the matrix of fried meat products. These chemical reactions lead to the production of harmful substances, such as trans fatty acids (TFAs), in fried meat products [26]. Studies have shown that excessive intake of TFAs promotes vascular inflammation and oxidative stress and accelerates the development of AS [27]. Numerous academic organizations have recommended that the intake of saturated fatty acids and TFAs should be limited to regulate blood lipid levels in high-risk populations [28]. Importantly, even though the potatoes that people in western China like to eat are a good source of carbohydrates [29], the frying cooking method leads to an increase in the risk of noninfectious diseases such as CVD and diabetes by affecting inflammatory factors and vascular endothelial function [30]. This showed that when designing a diet plan for patients with AS-related diseases in western China, the patients should be asked to limit their intake of fried, high-fat foods, even if they like to eat these foods.

Most participants took the initiative to adjust their diet after being diagnosed with the disease. Some participants indicated that they had actively chosen a vegetarian diet or consciously tended to eat vegetables and fruits. People in western China often use boiled water to cook vegetables when they choose to eat vegetables and form a local characteristic dish, “Suguadou”. Commonly consumed vegetable types included kidney beans, immature pumpkin. Studies have shown that the choice of cooking method is related to cardiovascular risk factors. In addition to raw food, boiling is also a healthier cooking method, which is related to healthier cardiovascular conditions [31]. Boiled cooking methods could also better retain antioxidant compounds in vegetables. We found that people in western China like to eat a seasonal fruit called RRT in summer. This fruit is a medicinal plant and traditional food in western China. In recent years, studies have shown that RRT is rich in vitamin C [32]. The presence of other substances (organic acids, flavonoids, polyphenols, etc.) can improve dyslipidaemia through the intestinal flora [33]. Therefore, eating RRT or drinking freshly squeezed fruit juice might improve AS-related diseases.

In addition, people in western China were also keen to eat “red sour soup”. “Red sour soup” is a common fermented seasoning in Guizhou Province, China. It is mainly fermented with “Maolaguo”, red peppers, etc., followed by the addition of Litsea cubeba fruit essential oil [34]. People often use “red sour soup” to cook vegetables, lean meat slices, fish slices and so on. Studies have shown that “red sour soup” can alleviate nonalcoholic fatty liver disease induced by a high-fat diet in rats and reduce body mass index, total cholesterol, triglyceride, and insulin resistance [35]. According to a study by Yang et al. [36], red sour soup can prevent and treat hyperlipidaemia in obese rats by regulating the AMPK signalling pathway, which might be related to the antioxidant and anti-atherosclerotic effects of lycopene and capsaicin, which are abundant among the red sour soup raw materials [37].

Studies have shown that the fermentation process of red sour soup will produce beneficial bacteria such as *Lactobacilli, Acetobacter*, and *Leuconostoc* and acid substances such as lactic acid, acetic acid and citric acid [38]. These acids regulate inflammation and promote immunity, neuroprotection, and anti-ageing activity [39]. However, the impact of food as a whole on the health of organisms rather than the impact of a single component of food [40] should be noted. Therefore, it is necessary to comprehensively consider the impact of red sour soup on human health; that is, the beneficial effects of red sour soup on human health are due to its rich bioactive substances and beneficial components produced during fermentation.

Notably, some male participants mentioned frequent consumption of alcohol. Studies have shown that higher alcohol intake increases the risk of CVD mortality in Chinese men and that alcohol intake does not have a protective effect on CVD [41]. Although participants might reduce or stop consuming alcohol after the diagnosis of AS-related diseases, past studies have shown that patients who continue to drink alcohol have a similar risk of death to those who have quit [42]. This suggested that the harm caused to the human body by alcohol consumption is permanent, even if the patient has chosen to quit drinking alcohol.

This study revealed that participants generally lack healthy eating knowledge. Research has shown that among participants, there is a widespread bias towards certain types of food and a misconception regarding nutritional assistance. A survey of elderly individuals [43] revealed similar findings; for example, some participants believed that “thin” is healthy and “fat” is unhealthy, and they believed that fat, sugar, etc., are “bad” foods and prefer vegetarian food [44]. However, studies have shown that proper fat intake is beneficial to human health, and people should consume a certain amount of high-quality fat and reduce saturated fat intake [45]. The intake of omega-3 fatty acids had some benefits for participants with cardiovascular and cerebrovascular diseases [46]. Many studies have shown that a plant-based diet can promote vascular endothelial protection and reduce the generation of harmful factors in endothelial cells, which is beneficial for treating AS-related diseases [47]. A meta-analysis of 55 studies showed that compared to other eating patterns, plant-based diets and whole-grain foods are associated with better prevention of coronary heart disease and multiple metabolic diseases [48]. However, it is worth noting that even though plant-based diets have been shown to be beneficial to human health, all dietary patterns are associated with potential nutritional risks [49]. Studies have shown that long-term intake of a vegan diet may lead to a lack of micronutrients, resulting in potential nutritional risks [50]. Therefore, for elderly patients with AS-related diseases, dietary guidance should include prompting patients to choose a balanced diet, consuming abundant plant-based foods, and correcting their misunderstanding regarding their current dietary patterns.

In contrast, there were also some participants who had received relevant health education, but the information provided by the internet may conflict with it, making it difficult for them to consume a healthy diet. Numerous studies have shown that the quality of health-related information that patients can learn on the internet is mixed [51]. Many sources of information were nonprofessionals who had not received medical professional training, which leads to mixed and inaccurate or biased information that may mislead patients and even have a negative impact on their health [52]. However, even if there was erroneous or unconfirmed information, viewing internet videos was still a popular method of health education for patients. Health education, in which professional people use networks, can significantly improve patients’ compliance behaviour and reduce costs [53]. However, in this survey, some participants were unable to obtain health knowledge by reading or watching videos because of old age, illness or disability. At the same time, some participants suggested that after leaving the medical environment, doctors or nurses could not guide and supervise their diet, which led them to collect relevant health knowledge in other ways, and their compliance behaviour gradually decreased over time. Doctors or nurses should carry out continuous and personalized health education for patients. Notably, only providing advice on improving diet and activity behaviours is not enough to change and maintain these behaviours in the long run. Effective health education that supports behavioural changes requires effective incentives and promotion, including environmental support [54], and provides patients with intervention methods suitable for their culture, age and other characteristics [55].

The majority of participants accepted nutritional assistance. Our survey showed that elderly participants with AS-related diseases need personalized nutritional assistance to improve their physical condition. In addition to the need for nutritional assistance, they also need corresponding dietary support from the government or institutions because the diseases limits their physical movement [56]. At the same time, because of the decline in functional living ability, many participants showed dependence on their families. This finding was consistent with most studies [57]. With the widespread promotion of medical-nursing combinations in China, meals are increasingly being prepared by medical-nursing combined institutions rather than by the patients themselves, community health service institutions, etc., to improve diet quality. Based on the patient interviews, we found that the nutritional assistance provided by medical-nursing combined institutions may be more suitable for and accepted by elderly patients with AS-related diseases. Medical-nursing combined institutions could help elderly people with full and partial disability to solve the problems of meals, medical treatment and self-care at a lower cost. In some European and American countries, there have been similar nutritional assistance models for elderly people, but most of them involve modelled nutrition management, such as communities providing three meals a day to elderly people in the form of meal boxes. However, this intervention model cannot be used for personalized service [58].

In contrast, some participants thought that they did not need to receive nutritional assistance. They held the mentality of ‘being so old’ and had a resistant and unacceptable attitude towards nutritional assistance. This might be because they think they were old enough to no longer have to put much effort into fighting the death caused by the diseases. This study revealed that elderly people with increasing age are becoming increasingly more deeply aware of the limitations of their lives. They could accept death as an inevitable event and reduce their avoidance of death [59]. However, it should be noted that the participants’ lack of healthy diet knowledge may have led them to mistakenly believe that diet cannot significantly improve the clinical manifestations of AS-related diseases, so they still maintain unhealthy eating habits and refuse to perform healthy lifestyles. Moreover, these participants might underestimate the consequences of poor lifestyles, resulting in serious cardiovascular events, including vascular obstruction and vascular rupture. These conditions might lead to paralysis, dysphagia and other symptoms, which would result in reduced or even loss of self-care ability and a significant reduction in quality of life [60].

This study has several limitations. The research team tried to recruit participants with heterogeneous characteristics, including age, sex, family status, and education level. However, due to the purposive sampling method, the results of this study may not be extended to the wider Chinese or international population of elderly patients with coronary and other atherosclerotic vascular diseases. This study excluded individuals who did not speak Chinese. Therefore, we cannot determine whether the samples of this study included multicultural or multiethnic groups.

## Conclusion

This study showed that elderly patients with coronary and other atherosclerotic vascular diseases who are living in western China have regional dietary preferences, which may have a certain impact on their disease development. They have different views due to differences in sex, disease status, personal habits, and modes of receiving dietary knowledge. These views are mainly regarding their own dietary status, cooking behaviours, and dietary management models. Regional and individual differences may influence the effects of diet management. In the future, for research regarding the dietary management of elderly patients with coronary and other atherosclerotic vascular diseases in western China, researchers should conduct personalized and sex-specific dietary management interventions according to their regional dietary preferences and consider whether individual patients are able to receive relevant nutritional assistance. Medical and nursing combination institutions can provide them with modelled nutrition management, such as providing three meals in the form of lunch boxes or open canteens. They can also use a variety of methods, such as face-to-face conversations and meetings, to provide them with dietary advice and flexibly use the internet to achieve online intervention. Changes in dietary behaviour may have a positive impact on the overall dietary quality of this population and may improve the patient’s disease status and prognosis.

### Supplementary Information

Supplementary Material 1.

## Data Availability

The datasets generated and/or analysed during the current study are not publicly available due ethical reasons but are available from the corresponding author on reasonable request.

## Abbreviations

AS: Atherosclerosis
CVD: Cardiovascular Disease
RRT: *rosa roxburghii Tratt*
TFA: Tras Fatty Acid
