# Supplementary material for: Cognition of diet quality and dietary management in elderly patients with coronary and other atherosclerotic vascular disease in western China, a qualitative research study
Source: BMC Geriatr. 2024 Jun 17;24:525. doi: 10.1186/s12877-024-05058-2 (PMC11184894; doi:10.1186/s12877-024-05058-2)
Supplement: Supplementary file 1 — Supplementary Material 1. [file 12877_2024_5058_MOESM1_ESM.zip › Appendix A.docx]

Appendix A

Interview Guide：

1.If you don 't mind, could you tell me something about your coronary and other atherosclerotic vascular disease ?

2.What do you think of your current diet ?

3.If patients think that their current diet needs to be improved : 1 Are there factors that prevent you from getting a better diet ? 2 What will help you eat better ?

4.What do you think your food intake can be improved ?

5.Has anyone talked to you about the nutritional problems associated with atherosclerosis ?

6.Do you want help with food intake ?

7.Now I will introduce some different types of nutritional assistance, you can choose your favorite way, and tell me the reasons to choose or not to choose.

1 A one-on-one interview with a dietitian to advise you on your diet. If you like this way, are you willing to go face-to-face or via telemedicine ?

2 Lectures, conferences, etc. hosted by dietitians. If you like this option, would you like to go face-to-face or via telemedicine ?

3 A prescription for a daily dietary supplement by a professional physician.

4 The meals are cooked by the community or the medical and nursing combination institutions and provided to you.

5 Recipes written by professionals are provided to you.

6 Assist you in making meal plans, writing shopping lists, and providing them to you.

With all of these options in mind, what 's your first choice, and what aspects of this option do you like ?

Do you have any additional comments ?

Thank you very much for your time today.
