# Supplementary material for: Cognition of diet quality and dietary management in elderly patients with coronary and other atherosclerotic vascular disease in western China, a qualitative research study
Source: BMC Geriatr. 2024 Jun 17;24:525. doi: 10.1186/s12877-024-05058-2 (PMC11184894; doi:10.1186/s12877-024-05058-2)
Supplement: Supplementary file 1 — Supplementary Material 1. [file 12877_2024_5058_MOESM1_ESM.zip › figure1 figure2_ESM.pdf]

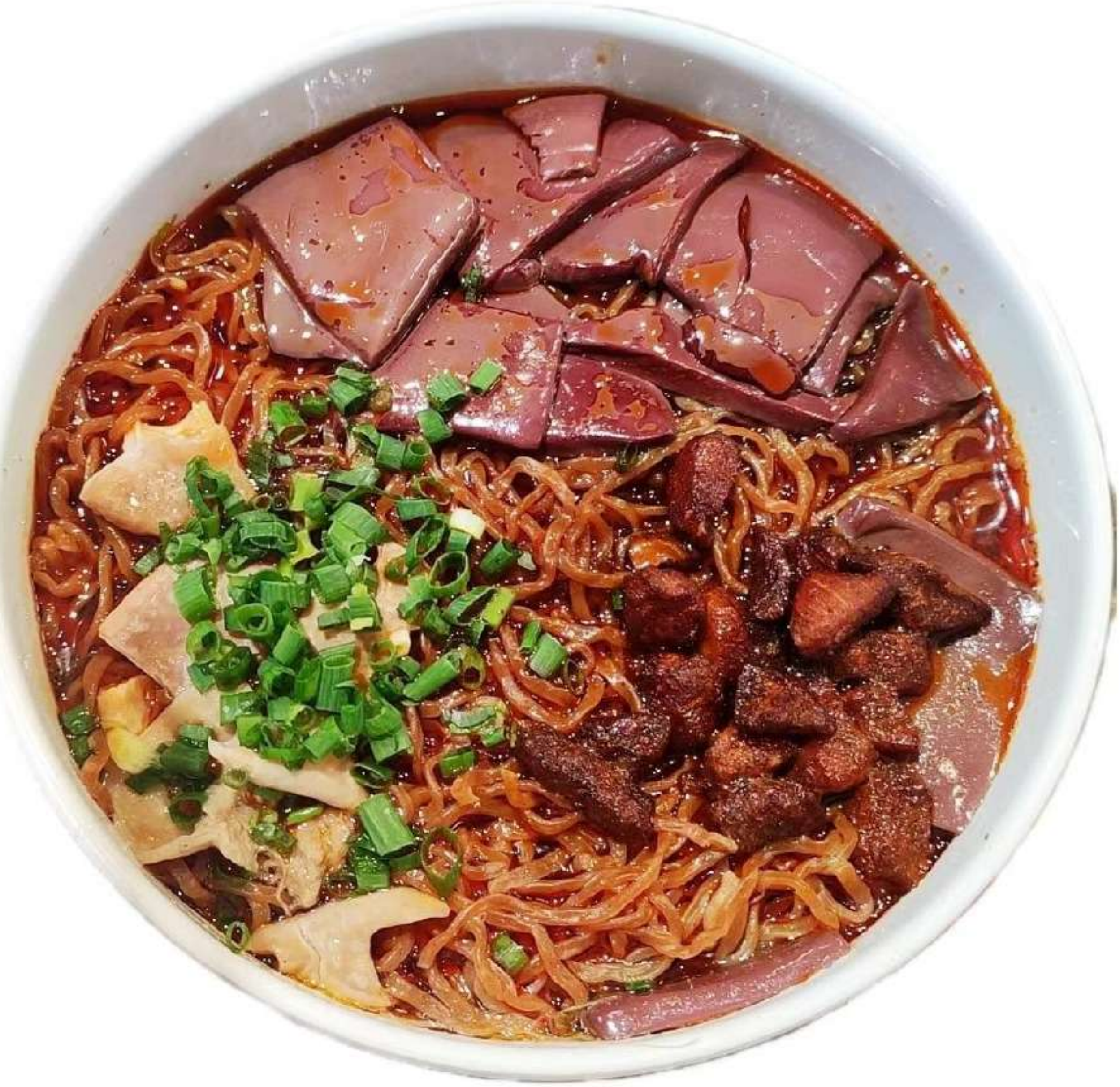

figure1: ChangWang noodles from Guizhou, China

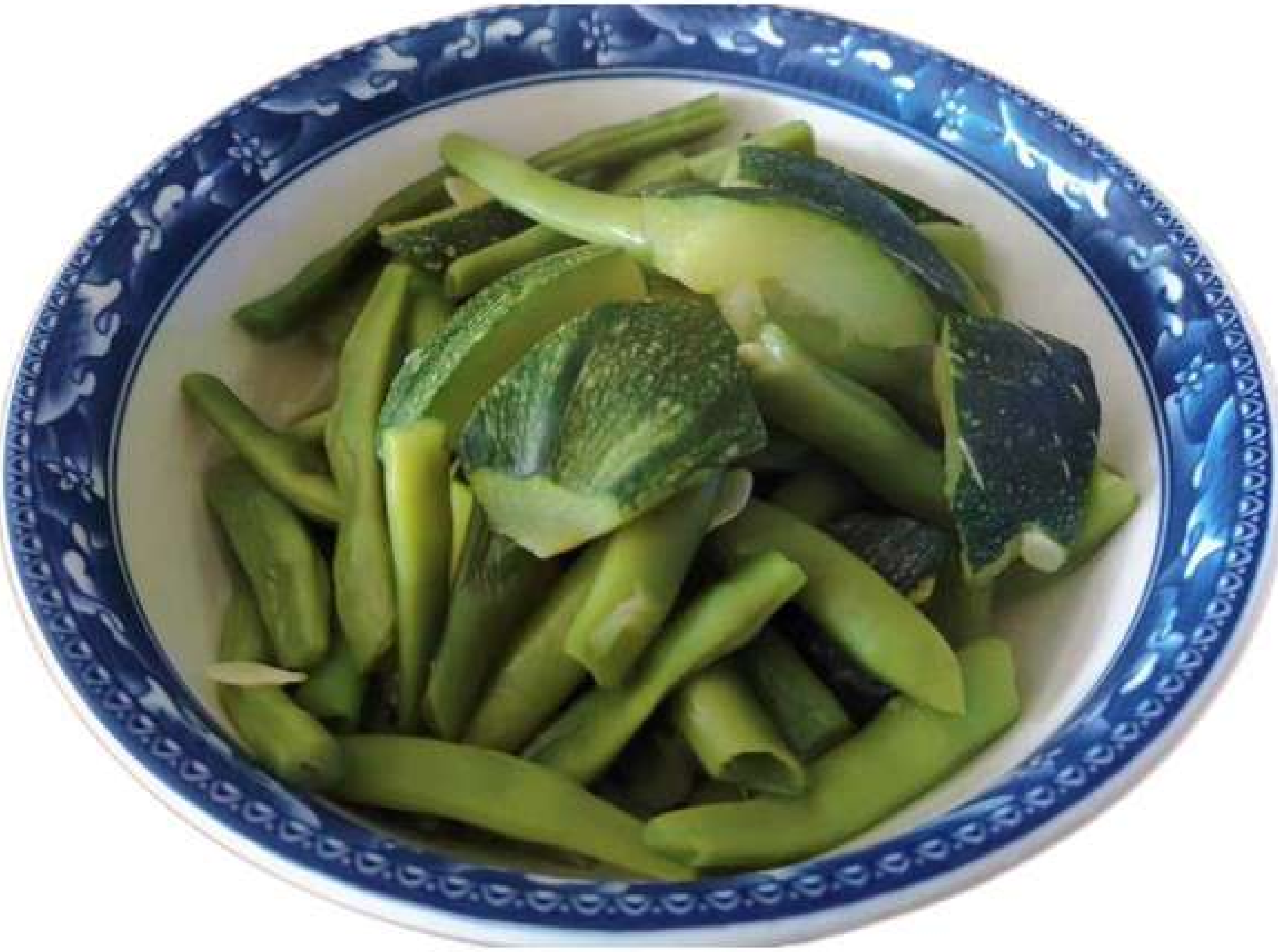

figure2: "Suguadou" included kidney beans and immature pumpkin
